# Pharmacokinetic targeting of intravenous busulfan reduces conditioning regimen related toxicity following allogeneic hematopoietic cell transplantation for acute myelogenous leukemia

**DOI:** 10.1186/1756-8722-3-36

**Published:** 2010-10-06

**Authors:** Joseph Pidala, Jongphil Kim, Claudio Anasetti, Mohamed A Kharfan-Dabaja, Taiga Nishihori, Teresa Field, Janelle Perkins, Lia Perez, Hugo F Fernandez

**Affiliations:** 1 Department of Blood and Marrow Transplantation, Moffitt Cancer Center, 12902 Magnolia Drive, Tampa, FL, 33612, USA; 2 Department of Biostatistics, Moffitt Cancer Center, 12902 Magnolia Drive, Tampa, FL, 33612, USA; 3 Department of Oncological Sciences, University of South Florida, Tampa, FL 33612, USA

## Abstract

Optimal conditioning therapy for hematopoietic cell transplantation (HCT) in acute myelogenous leukemia (AML) remains undefined. We retrospectively compared outcomes of a consecutive series of 51 AML patients treated with oral busulfan (1 mg/kg every 6 hours for 4 days) and cyclophosphamide (60 mg/kg IV × 2 days) - (Bu/Cy) with 100 consecutive AML patients treated with pharmacokinetic targeted IV busulfan (AUC < 6000 μM/L*min per day × 4 days) and fludarabine (40 mg/m2 × 4 days) - (t-IV Bu/Flu). The Bu/Cy and t-IV Bu/Flu groups significantly differed according to donor relation, stem cell source, aGVHD prophylaxis, remission status, primary vs. secondary disease, median age, and % blasts prior to HCT (p < 0.01 for each). Conditioning with t-IV Bu/Flu reduced early toxicity including idiopathic pneumonia syndrome (IPS) and hepatic veno-occlusive disease (VOD). Additionally, the trajectory of early NRM (100 day: 16% vs. 3%, and1 year: 25% vs. 15% for Bu/Cy and t-IV Bu/Flu, respectively) favored t-IV Bu/Flu. Grade II-IV aGVHD (48% vs. 82%, p < 0.0001), as well as moderate/severe cGVHD (7% vs. 40%, p < 0.0001) differed between the Bu/Cy and t-IV Bu/Flu groups, due to the predominance of peripheral blood stem cells in the t-IV Bu/Flu group. Pharmacokinetic targeting of intravenous busulfan in combination with fludarabine is associated with reduced conditioning regimen related toxicity compared to oral busulfan and cyclophosphamide. However, multivariable analysis did not demonstrate significant differences in overall survival (p = 0.78) or non-relapse mortality (p = 0.6) according to conditioning regimen delivered.

## Background

Ongoing investigation aims to preserve efficacy, but reduce morbidity and mortality associated with conditioning therapy for allogeneic hematopoietic cell transplantation (HCT). Seminal work has demonstrated variation in bioavailability of oral busulfan (Bu), and the correlation between busulfan exposure and both toxicity including hepatic veno-occlusive disease,[1] as well as graft rejection and primary disease relapse.(8, 9) In the setting of both oral and intravenous administration of busulfan, inter-patient variation is observed[2]. Importantly, the intended busulfan exposure differs according to individual transplantation conditioning regimens, such as Bu/Cyclophosphamide(Cy) and Bu/Fludarabine(Flu). As well, in the context of Cy/total body irradiation(TBI) based conditioning therapy, *McDonald, et al *have demonstrated increased non-relapse mortality and reduced overall survival associated with exposure to toxic metabolites of cyclophosphamide[3]. Conversely, fludarabine given in combination with targeted oral [4] or intravenous busulfan[5,6] has been demonstrated safe and effective as conditioning therapy for HCT in myeloid malignancies.

With the intention of reducing transplant related toxicity, as well as expanding access to patients of older age or more advanced comorbidity, a number of reduced to intermediate-intensity, or truly non-myeloablative regimens have been developed[4,5,7-15]. Our center adopted an approach of pharmacokinetic-targeted IV Bu/Flu from 2004 onward as a uniform conditioning strategy in the setting of allogeneic transplantation for acute myelogenous leukemia. As the regimen appears to result in reduced treatment related toxicity compared to our center's historical experience with oral busulfan and cyclophosphamide, we aimed to confirm these observations in a comparative analysis of outcomes of AML patients.

## Methods

### Patients

All consecutive acute myelogenous leukemia (AML) patients treated with a conditioning regimen of targeted IV busulfan and fludarabine (t-IV Bu/Flu) at Moffitt Cancer Center from 2004 to 2008 with a minimum follow up of one year were identified. These were compared to a consecutive historical cohort of 51 consecutive AML patients treated with oral Busulfan and Cyclophosphamide (Bu/Cy) at the Center from 1997 to 2004. These data demonstrate a trend by which our center's volume of allogeneic transplants for AML have substantially increased over this time frame. As well, supportive care practices and program faculty have changed over this time frame. All patients provided informed consent for follow up of transplant outcome data. The reporting of this data was approved by the University of South Florida Institutional Review Board.

### Conditioning, GVHD prophylaxis, and supportive care

The conditioning regimen in the t-IV Bu/Flu group consisted in all cases of Fludarabine, 40 mg/m^2 ^infused over 30 minutes daily on days -6 to -3, followed by intravenous Busulfan, 130-145 mg/m2 over 4 hours daily on the same days. Busulfan (BU) pharmacokinetic samples were obtained on day -6 and analyzed by mass spectrometry in the clinical toxicology lab at the University of Pennsylvania. Analysis utilized a one compartment model with first order kinetics. On days -4 and -3, the BU dose was adjusted to target an average AUC of 5300 (+/-10%) μMol*min (n = 96) or 3500 (+/-10%) μMol*min (n = 4) for each of the four days. The lower target AUC of 3500 was chosen in these 4 cases due to patient age, ranging from 62 to 67. The Bu/Cy regimen consisted in all cases of oral busulfan 1 mg/kg every 6 hours for 4 days (days -7 to -4) for a total of 16 doses, as well as cyclophosphamide 60 mg/kg IV × 2 days (days -3 to -2). Pharmacokinetic measurements were not performed in this group. Stem cell source and GVHD prophylaxis are reported in table 1.

**Table 1:** Baseline and transplant characteristics of patients in Bu/Cy and t-IV Bu/Flu groups

Variables		Bu/Cy (Freq/Percent)	Bu/Flu (Freq/Percent)	Total (Freq)	P-value
Name	Level				
**Time from diagnosis to HCT**	**Months (median value, range)**	6.2 (1-29)	6.4 (2-54)		0.5
**Follow up time**	**Months (median value, range)**	8 (1-90)	17 (1-53)		0.245
**Donor**	**MRD**	51 100.00	39 39.00	81	< .0001
	**MUD**	0 0.00	39 39.00	39	
	**MMUD**	0 0.00	22 22.00	22	
**Cytogenetic**	**Low**	3 7.89	7 7.00	10	0.3568
	**Intermediate**	25 65.79	54 54.00	79	
	**High**	10 26.32	39 39.00	49	
**ATG**	**No**	42 100.00	78 78.79	120	0.0006
	**Yes**	0 0.00	20 20.20	20	
**Cell Source**	**BM**	35 68.63	2 2.00	37	< .0001
	**PB**	16 31.37	98 98.00	114	
**aGVHD prophylaxis**	**CSA/MTX**	51 100.00	0 0.00	42	< .0001
	**TAC/MMF**	0 0.00	22 22.00	22	
	**TAC/MTX**	0 0.00	77 71.00	77	
	**TAC/RAPA**	0 0.00	1 1.00	1	
**Disease Status**	**CR1**	19 37.25	49 49.00	68	0.0013
	**CR2 or CR3**	6 11.76	25 25.00	31	
	**PIF**	7 13.73	16 16.00	23	
	**Relapse**	18 35.29	9 9.00	27	
	**UNT**	1 1.96	1 1.00	2	
**Diagnosis**	**primary**	41 80.39	62 62.00	103	0.0221
	**secondary**	10 19.61	38 38.00	48	
**No of Induction**	**0**	1 2.38	1 1.00	2	0.4882
	**1**	31 73.81	67 67.00	98	
	**2**	9 21.43	24 24.00	33	
	**3**	1 2.38	8 8.00	9	
**AGE**		39.0 (19.6-55.60)	48.16(21.84-68.64)		0.0001
**WBC at Diagnosis**		13.60 (0.80-190.00)	5.80 (0.25-285.00)		0.1073
**Time in CR1**		256 (12-762)	324.5 (21 - 2679)		0.1699
**% Blast in BM**		4 (0 - 88)	2 (0-80)		0.0028
**Chimerism (day 90)**	**BM**	100 (55-100)	97 (10-100)		0.0925
	**CD3**	NA	90 (18-100)		NA
	**CD33**	NA	100 (10-100)		NA

### Outcomes

Neutrophil engraftment was defined by the first of three successive days with an absolute neutrophil count of greater than 500/uL. Platelet engraftment was defined by the first of three successive days with a non-transfused platelet count of greater than 20,000/uL. The occurrence and severity of hepatic sinusoidal obstructive syndrome (veno-occlusive disease) were recorded using the previously described criteria[16,17]. Acute graft vs. host disease (aGVHD) was scored per modified Glucksberg criteria[18]. Chronic graft vs. host disease (cGVHD) was scored per proposed NIH consensus criteria[19]. Peripheral blood sorted (CD3 and CD33) and bone marrow donor chimerism were assessed by PCR. Primary disease restaging occurred in all cases at minimum on days 30, 90, 180, and 360, as well as at 18 months, and 2 years.

### Statistical analysis

Differences in baseline characteristics were compared with Wilcoxon rank-sum test for numerical or ordinal variables, and Chi-square test or Fisher exact test for categorical variables. Time to neutrophil and platelet engraftment was calculated using the Kaplan-Meier method; comparison was made with log-rank test. Overall survival (OS) and progression-free survival (PFS) were estimated from date of transplantation using the Kaplan-Meier method; death or relapse was considered an event in the estimation of PFS. Accounting for competing risk events, the cumulative incidence (CI) of relapse and non-relapse mortality (NRM) was calculated by the Gray method[20]. For OS and PFS, baseline variables were examined with univariable, and Cox proportional hazard modeling. For CI of relapse and NRM, a sub-distribution hazards regression model was utilized for univariable and multivariable analysis[21]. For each outcome, distinct models were created to examine pre- and separately post-HCT variables. Pre-HCT variables considered included the following: conditioning regimen, cytogenetic risk group, donor relation, remission status, number of induction cycles for CR1 patients, stem cell source, GVHD prophylaxis regimen, primary vs. secondary AML, % blasts in bone marrow immediately prior to HCT, white blood cell count at diagnosis of AML, age at time of HCT, and time in CR1 for those who were transplanted in CR2/3 or with relapsed disease. Post-HCT variables considered included the following: maximum grade aGVHD, maximum grade cGVHD, donor chimerism in bone marrow at day 90 post-HCT, and donor CD3 and CD33 chimerism in peripheral blood on day 90 post-HCT. Those variables with p value of 0.25 or less in univariable analysis were selected for construction of the multivariable model. The backward selection procedure with a p-out value of 0.1 was utilized.

## Results

One hundred consecutive adults with AML received t-IV Bu/Flu, and 51 consecutive AML patients received oral busulfan without PK targeting and cyclophosphamide (Bu/Cy). The t-IV Bu/Flu conditioning regimen incorporated fludarabine, 40 mg/m^2 ^infused over 30 minutes on days -6 to -3, then intravenous busulfan, 130-145 mg/m2 over 4 hours daily on the same days. Busulfan (Bu) PK-samples were obtained on day -6 and analyzed by mass spectrometry; the BU dose was adjusted on days -4 and -3 to achieve an overall average AUC of 5300 (+/-10%) μMol*min (n = 96) or 3500 (+/-10%) μMol*min (n = 4) for each of the four days. The median actual AUC after the first dose was 5113 μM*min (range 2796 - 9355) for the 5300 μM*min target subgroup and 4244 uM*min (range 2830 - 5347) for the 3500 μM*min target subgroup. Daily busulfan doses were adjusted to achieve the target AUC averaged over 4 days. Median total BU dose required to achieve the target AUC was 520 mg/m^2 ^(range 370 - 974) in the 5300 μM/L*min group and 418 mg/m^2 ^(range 254 - 470) in the 3500 μM/L*min group. Baseline characteristics are summarized in table 1. Importantly, there were significant differences across the following baseline characteristics in the Bu/Cy and t-IV Bu/Flu groups: donor relation, stem cell source, aGVHD prophylaxis, remission status, primary vs. secondary disease, median age, and % blasts prior to HCT.

### Comparison of Bu/Cy vs. t-IV Bu/Flu: composite groups

**Figure 1:**
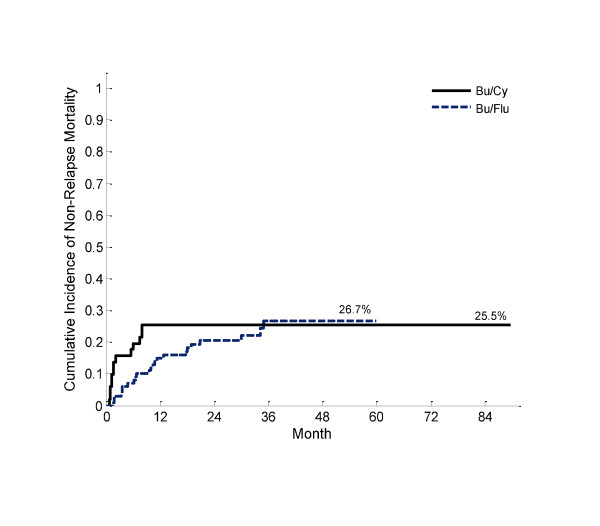
**Cumulative incidence of NRM according to conditioning regimen (p = 0.65)**.

Median time to neutrophil engraftment was 18 vs. 16 days (p < 0.001), and median time to platelet engraftment was 21 vs. 12 days (p = 0.0002) for Bu/Cy and t-IV Bu/Flu, respectively. Differences in engraftment are attributable to the preponderance of peripheral blood stem cells (PBSC) in the t-IV Bu/Flu group. There was a greater burden of conditioning regimen related toxicity observed after Bu/CY: There were 5 fatal cases of IPS and 6 cases (mild 1; moderate 4; and severe, fatal case 1) of VOD after conditioning with Bu/Cy. Conversely, in the t-IV Bu/Flu group there was one case of idiopathic pneumonia syndrome (IPS) and no cases of hepatic sinusoidal obstructive syndrome (VOD). While the ultimate CI of non-relapse mortality (NRM) did not significantly differ between groups (figure 1), early NRM was reduced in the t-IV Bu/Flu group (table 2).

**Table 2:** Non-relapse mortality according to treatment regimen

	t-IV Bu/Flu	Bu/Cy
100 days	3%	16%
6 months	7%	18%
1 year	15%	25%

There were significant differences in grade II-IV aGVHD (48% vs. 82%, p < 0.0001), as well as moderate/severe cGVHD (7% vs. 40%, p < 0.0001) between the Bu/Cy and t-IV Bu/Flu groups, respectively; the marked increase in the incidence of GVHD observed in the t-IV Bu/Flu arm is attributable to the greater proportion of PBSC. The disparity in NRM in the first year after HCT suggests less conditioning regimen toxicity after t-IV Bu/Flu. However, NRM trends toward convergence over 1 and 2 years, likely secondary to this increased risk of GVHD.

**Figure 2:**
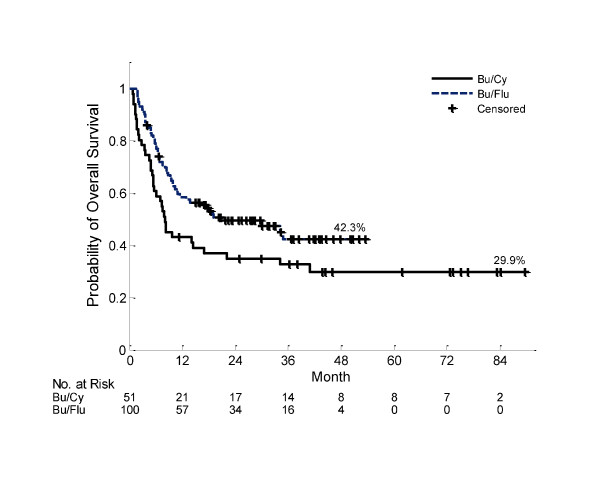
**Overall survival for total sample in Bu/Cy and t-IV Bu/Flu groups (log-rank p = 0.0356)**.

With median follow up for living patients of 54 months (range 11 - 90) for Bu/CY and 28 months (range CI 2 - 54) for t-IV Bu/Flu, median OS was 8 months (95% CI 5.4 - 22) vs. 21 months (95% CI 10.9 - not reached), respectively for each group (figure 2). Causes of death for Bu/Cy were: infection (n = 4), relapse (n = 22), idiopathic pneumonia syndrome (n = 5), hepatic VOD (n = 1), multi-system organ failure (n = 1), unknown (n = 1), and TTP/HUS (n = 1). Causes of death in the t-IV Bu/Flu group included: refractory aGVHD (n = 4), refractory cGVHD (n = 1), infection (n = 7), relapse (n = 29), multi-organ system failure (n = 7), post-transplant lymphoproliferative disorder (n = 1), and unknown (n = 3). Median PFS was 6.9 months (95% CI 4.1 - 20) in the Bu/Cy group vs. 15.1 months (95% CI 9.1 - 30) in the t-IV Bu/Flu group. There was no significant difference in the cumulative incidence of AML relapse at day 100 (16% vs. 17%), 1 year (33% vs. 32%), or 2 years (40% vs. 37%) after HCT in the comparison of Bu/Cy vs. t-IV Bu/Flu, respectively, p = 0.68.

### Comparison Bu/Cy vs. t-IV Bu/Flu according to disease risk subgroups

#### Complete remission 1 (CR1)

**Figure 3:**
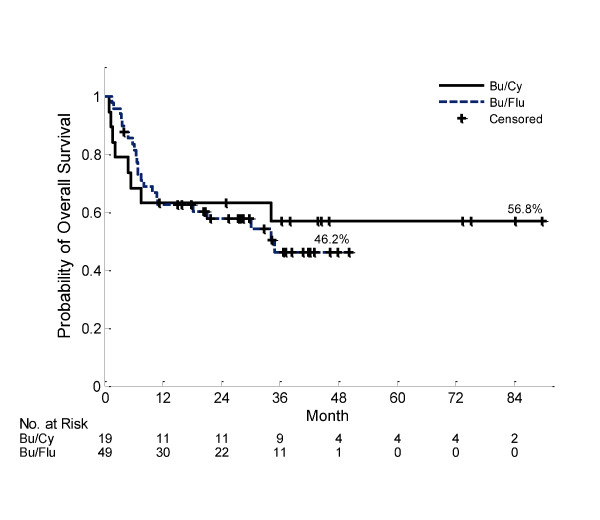
**Overall survival for those in CR1 at time of HCT, stratified by conditioning regimen (log-rank p = 0.78)**.

Nineteen patients received Bu/Cy in CR1, and 49 received t-IV Bu/Flu in CR1. The proportion with primary vs. secondary AML in CR1 (Bu/Cy: primary n = 13, secondary n = 6; t-IV Bu/Flu: primary n = 30, secondary n = 19, Fisher exact p = 0.78) and cytogenetic risk group (Bu/Cy: low n = 0, intermediate n = 10, high n = 3, n/a n = 6; t-IV Bu/Flu: low n = 2, intermediate n = 26, high n = 21, Fisher exact p = 0.28) did not significantly differ between the Bu/Cy and t-IV Bu/Flu groups. There was no significant difference in OS between these groups (1 year OS 63% vs. 63%, and 2 year OS 63% and 58% for Bu/Cy vs. t-IV Bu/Flu, p = 0.78) respectively (figure 3). There was also no significant difference between groups for those with primary AML in CR1 (1 year OS 69% vs. 66%, 2 year OS 69% vs. 62% for Bu/Cy vs. t-IV Bu/Flu, p = 0.8) and those with secondary AML in CR1 (1 year OS 69% vs. 66%, 2 year OS 69% vs. 62% for Bu/Cy vs. t-IV Bu/Flu, p = 0.9). As well, there was a non-significant increase in early non-relapse mortality in the Bu/Cy group (100 day 21% vs. 4%, 1 year 26% vs. 16%, and 2 year 26% vs. 21%, p = 0.98).

#### Complete remission 2 or 3 (CR2/3)

Six patients received Bu/Cy conditioning in CR 2/3 and 25 patients received t-IV Bu/Flu conditioning in CR 2/3. OS was significantly worse in those in the Bu/Cy group (1 year OS 17% vs. 59%, 2 year OS 17% vs. 41% for Bu/Cy vs. t-IV Bu/Flu, p = 0.03). NRM (100 day 0% vs. 0%, 1 year 50% vs. 16%, and 2 years 50% vs. 24% for Bu/Cy vs. t-IV Bu/Flu, p = 0.14) did not significantly differ.

#### Primary induction failure (PIF)

Seven patients received Bu/CY as conditioning in the setting of primary induction failure, and 16 patients received t-IV Bu/Flu. OS significantly differed (1 year OS 29% vs. 69%, 2 year OS 14% vs. 61% for Bu/Cy vs. t-IV Bu/Flu, p = 0.01) between groups. NRM (100 day 0% vs. 0%, 1 year 14% vs. 6%, and 2 year 14% vs. 6% for Bu/Cy vs. t-IV Bu/Flu, p = 0.58) did not significantly differ.

### Refractory relapsed disease

Eighteen patients with refractory relapsed AML were treated with Bu/Cy, and 9 received t-IV Bu/Flu. There was no significant difference in OS (1 year OS 33% vs. 22%, 2 year OS 17% vs. 0% for Bu/Cy vs. t-IV Bu/Flu, p = 0.52). NRM (100 day 22% vs. 11%, 1 year 22% vs. 22%, and 2 year 22% vs. n/a for Bu/Cy vs. t-IV Bu/Flu, p = 0.61) did not significantly differ between groups.

### Multivariable modeling

Baseline pre-transplant variables were first considered in univariable analysis to evaluate the relationship of each with OS. In the multivariable model, only relapsed disease at time of HCT remained a significant predictor of OS, HR 3.3 (95% CI 1.4 - 7.9; p = 0.007). Conditioning regimen did not significantly predict OS. In multivariable analysis of post-HCT variables, moderate/severe cGVHD - HR of 0.4 (95% CI 0.18 - 0.88; p = 0.02) - and day 90 bone marrow chimerism ≥ 90% - HR of 0.28 (95% CI 0.11 - 0.71; p = 0.008) - predicted OS.

Baseline variables were also examined in univariable analysis to discern their relationship with progression-free survival. On construction of a multivariable model using pre-HCT variables, remission status emerged as an independent predictor of PFS. In reference to CR1, refractory relapsed disease predicted significantly worse PFS, with HR 3.0 (95% CI 1.3 - 7; p = 0.01). Conditioning regimen did not significantly predict PFS. Examination of post-HCT variables in multivariable modeling identified day 90 BM chimerism ≥ 90% as a significant predictor: HR 0.18 (95% CI 0.08 - 0.42; p < 0.0001).

In multivariable analysis of pre-HCT variables, relapse was significantly predicted by remission status: PIF (HR 3.0, 95% CI 1.2 - 7.5, p = 0.015) and refractory relapsed disease (HR 3.8, 95% CI 1.4 - 10.3, p = 0.009) were associated with significantly greater risk of relapse compared to a reference of CR1. Conditioning regimen did not significantly predict relapse. Of post-HCT variables, multivariable modeling identified day 90 BM chimerism ≥ 90% as a protective factor (HR 0.16, 95% CI 0.08 - 0.35, p < 0.001).

Finally, of pre-HCT variables, peripheral blood stem cells were associated with less NRM (HR 0.34, 95% CI 0.12 - 0.98, p = 0.046), as compared to a reference of bone marrow stem cells in multivariable analysis. Additionally, with reference of TAC/MMF aGVHD prophylaxis, both CSA/MTX (HR 0.18, 95% CI 0.05 - 0.63, p = 0.01) and TAC/MTX (HR 0.41, 95% CI 0.18 - 0.93, p = 0.03) predicted significantly lower NRM on multivariable modeling. There was a trend toward decreased NRM in the t-IV Bu/Flu group (HR 0.83, 95% CI 0.41 - 1.7, p = 0.6) compared to Bu/Cy in univariable analysis, which did not remain a significant predictor of NRM on multivariable modeling. Of post-HCT variables examined in uni- and multi-variable analysis, grade III/IV aGVHD demonstrated a non-significant trend toward greater NRM (HR 2.7, 95% CI 0.99 - 7.2, p = 0.053), as compared to a reference of grade 0/I.

## Discussion

Recognizing the adverse outcomes potentiated by variation in bioavailability of oral busulfan, as well as increased toxicity from aberrant metabolism of cyclophosphamide, there was a shift in the institutional practice at our center, wherein those consecutive AML patients transplanted from 2004 onward received intravenous PK-targeted busulfan combined with fludarabine (t-IV Bu/Flu) as conditioning therapy. We aimed to compare these outcomes to a historical cohort of AML patients, who received Bu/Cy as conditioning therapy in a single institution.

The most consistent finding from this analysis is the reduction in conditioning regimen related toxicity as well as reduction in early transplant related mortality with the adoption of t-IV Bu/Flu. A near elimination of severe busulfan toxicity including idiopathic pneumonia syndrome and hepatic veno-occlusive disease represents a major advantage in early HCT outcomes favoring t-IV Bu/Flu. These data corroborate the reduction in conditioning regimen toxicity anticipated with both pharmacokinetic targeting of busulfan, and avoidance of cyclophosphamide and it metabolites[3]. As well, while the ultimate cumulative incidence of NRM did not significantly differ between the t-IV Bu/Flu and Bu/Cy groups, the trajectory of NRM differed with less NRM at day 100 and 1 year in the t-IV Bu/Flu group. The significantly greater burden of aGVHD and severe cGVHD in the t-IV Bu/Flu group, explained by the predominance of peripheral blood stem cells, burden of unrelated and mismatched unrelated donors, and older age, resulted in the later approximation of these NRM curves. We acknowledge that these data on trends in early NRM and GVHD related outcomes are likely dependent upon the predominance of peripheral blood stem cells in the t-IV Bu/Flu group.

Of the 100 AML patients treated with t-IV Bu/Flu and 51 treated with Bu/Cy, there were important differences in baseline variables which circumscribe other comparisons. First, transplant conditions differed, which complicates observed differences in non-relapse mortality between groups: While the Bu/Cy group only included matched sibling donors, the t-IV Bu/Flu group included matched sibling donors, but the majority (61%) were either unrelated or mismatched unrelated donors. This in particular, as well as the significantly increased age and predominance of peripheral blood stem cells (98% of subjects) in the t-IV Bu/Flu group likely account for the significantly increased grade II-IV aGVHD and moderate/severe cGVHD realized in the t-IV Bu/Flu group. Second, the Bu/Cy and t-IV Bu/Flu groups differ according to disease risk variables, with a greater proportion of refractory relapsed disease at HCT in the Bu/Cy group, and conversely a greater proportion with secondary AML in the t-IV Bu/Flu group. The net effect of these disparate transplant and disease risk variables is difficult to discern. Accordingly, we have reported outcomes according to remission status, and also examined these variables in uni- and multi-variable modeling to discern the impact of conditioning regimen on outcome.

Acknowledging differences in disease risk variables between groups as well as the predominant use of PBSC in the t-IV Bu/Flu group, these results do not demonstrate significant differences in disease control between the two approaches. The cumulative incidence of relapse observed across groups both in the overall sample, as well as for each remission status subgroup, was similar. As well, conditioning regimen was not significantly associated with relapse on multivariable modeling. Specific conclusions regarding the impact of conditioning regimen on disease control among specific remission status groups are limited by small sample sizes. The most important predictor of relapse post-HCT in this series of AML patients was remission status at the time of HCT.

In total, these results demonstrate a reduction in conditioning regimen related toxicity and mortality after t-IV Bu/Flu for HCT in a consecutive series of adult AML patients. Previously published retrospective comparisons between comparable regimens of IV Bu/Flu and Bu/Cy have also demonstrated reduced toxicity in keeping with the conclusions of our analysis. In a retrospective analysis, *Andersson, et al *compared IV Bu/Flu and IV Bu/Cy2 as conditioning therapy for AML and MDS; groups differed significantly, with older age and a greater proportion of unrelated donors in the IV Bu/Flu group. Overall survival was significantly better in the IV Bu/Flu group[22]. *Bredeson, et al *compared IV Flu/Bu/ATG to oral Bu/Cy in a matched pair analysis compared to registry data including a heterogeneous array of hematologic malignancies; the Flu/Bu/ATG group had significantly older age, worse performance status, greater proportion of PBSC, and was transplanted in a later time period. This analysis demonstrated significantly decreased NRM in the Flu/Bu/ATG group[23]. Finally, *Chae, et al *compared IV Bu/Flu and oral Bu/Cy2; patient age was significantly greater in the IV Bu/Flu group. NRM was lower, and overall survival was significantly greater in the IV Bu/Flu group[24]. Overall, these retrospective comparisons suggest a significant advantage in transplantation outcomes favoring IV Bu/Flu. Randomized clinical trial data is needed to determine the true benefit of IV Bu/Flu over Bu/Cy conditioning for allogeneic HCT in acute myelogenous leukemia.

## Competing interests

The authors report the following funding sources which have relevance to the work described here: Claudio Anasetti, MD, and Janelle Perkins, PharmD have research funding from Protein Design Labs BioPharma for the conduct of research involving busulfan and fludarabine as conditioning therapy prior to allogeneic transplantation.

## Authors' contributions

All authors have read and approved this manuscript. JP designed the project, collected data, analyzed data, and wrote the manuscript; JK contributed to data analysis; CA contributed to design of project, analysis, and critical review of manuscript; MKD contributed to analysis and critical review of manuscript; TN contributed to analysis and critical review of manuscript; TF contributed to analysis and critical review of manuscript; JP contributed to analysis and critical review of manuscript; LP contributed to analysis and critical review of manuscript; and HF contributed to design of project, analysis, and critical review of manuscript.
